# *FAE1* and *FAD2* gene expression dynamics and fatty acid modulation in *Brassica* under salt stress: A molecular insight

**DOI:** 10.1371/journal.pone.0345945

**Published:** 2026-04-06

**Authors:** Samia Fatima, Nadia Iqbal, Shengqiao Lu, Muhammad Omar Khan, Muhammad Aamer Mehmood, Sameer H. Qari, Atif Jamal, Zhengyi Wei, Niaz Ahmad

**Affiliations:** 1 National Institute for Biotechnology and Genetic Engineering College, Pakistan Institute of Engineering and Applied Sciences (NIBGE-C, PIEAS), Faisalabad, Pakistan; 2 Maize Research Station, Ayub Agriculture Research Institute, Faisalabad, Pakistan; 3 Maize Research Institute, Guangxi Academy of Agricultural Sciences, Nanning, China; 4 Department of Bioinformatics & Biotechnology, Government College University Faisalabad, Faisalabad, Pakistan; 5 Biology Department, Al-Jumum University College, Umm Al-Qura University, Makkah, Saudi Arabia; 6 Crop Diseases Research Institute, National Agricultural Research Centre, Islamabad, Pakistan; Universidade de Coimbra, PORTUGAL

## Abstract

*Brassica* species are major oilseed crops valued for their high seed oil content; however, salinity severely limits their growth, productivity, and oil quality. Since membrane stability and oil composition are primarily governed by fatty acid metabolism, this study focused on two key lipid biosynthesis genes, *FAE1* (*Fatty Acid Elongase 1*) and *FAD2* (*Fatty Acid Desaturase 2*), to elucidate their role under NaCl-induced stress. Understanding the regulatory and functional behavior of these genes under salinity is essential for developing salt-resilient *Brassica* cultivars capable of maintaining oil quality on saline soils. To establish a mechanistic foundation, gene and protein sequences of FAE1 and FAD2 were retrieved from publicly available genomic databases. Various bioinformatics analyses were conducted to identify conserved domains, structural integrity, phylogenetic relationships, and regulatory features, thereby verifying their functional relevance in fatty acid biosynthesis. Promoter regions were also examined for stress-responsive cis-acting elements, including dehydration-responsive elements (DRE) and ABA-responsive elements (ABRE), providing predictive evidence of transcriptional regulation under salt stress. Based on these computational predictions, controlled salt stress was imposed to experimentally evaluate gene responsiveness and physiological adaptation. Tissue-specific expression analysis revealed significant differential regulation of *FAE1* and *FAD2* under salt stress, with pronounced transcriptional modulation observed in developing siliques and leaves compared to roots. Salt stress caused a dose-dependent reduction in growth attributes and yield-related traits in both species. Two-way ANOVA revealed significant NaCl effects on yield parameters (*F* = 6.6–20, *P* < 0.05), with seed yield declining by approximately 25–35% at 200 mM NaCl, with comparatively higher yield stability in *B. juncea* than in *B. napus* under salinity. Seed quality analysis showed species-specific responses in oil and protein content, whereas most fatty acids, including erucic acid, remained relatively stable. Despite transcriptional modulation of both genes, the overall fatty acid profile exhibited limited variation, suggesting metabolic buffering within the lipid biosynthetic network. Together, this integrative approach links *in silico* predictions with experimental validation, establishing a continuum from gene structure and regulation to phenotypic performance and seed quality. These findings enhance our understanding of lipid metabolism–mediated salt stress adaptation and provide a foundation for breeding and genome-editing strategies aimed at improving oilseed stability in *Brassica* under saline conditions.

## Introduction

The family *Brassicaceae* encompasses a wide range of diverse plant species, including oilseeds, vegetables, ornamentals, and condiments [1,2]. Amongst these, genus *Brassica,* comprising six species, has gained importance worldwide because of their diverse utility in food, animal feed, and industrial sectors [3,4]. This *Brassica* complex comprises three diploid species, i.e., *B. nigra*, *B. oleracea,* and *B. rapa*, and three amphidiploid species, i.e., *B. carinata*, *B. juncea,* and *B. napus* [5]. *B. juncea* and *B. napus* are major oilseed crops especially in the northern hemisphere, valued for their high oil content of about 40–48% [6]. The oil extracted from *B. napus*, especially from Canola-quality cultivars, is recognized as one of the healthiest cooking oils extensively used for edible purposes while *B. juncea* oil contains high amounts of erucic acid, making it more suitable for industrial uses rather than human consumption [7]. The remaining seed meal after extraction is a rich source of protein (38–40%) with a favourable amino acid profile, making it a nutritious feed for livestock [8].

However, the cultivation and utilization of these species present some advantages and limitations. For example, *B. napus* is a relatively delicate crop, more susceptible to biotic and abiotic factors such as insect and disease attack, salinity, heat, drought, and shattering. Despite these poor agronomic characteristics, it generally produces nutritionally superior edible oil and protein-rich animal feed [9–11]. In contrast, *B. juncea* is stress-resilient, better adapted to arid environments, demonstrates vigorous seedling development, faster ground covering ability, and more resilience to biotic (disease, insect) and abiotic (heat, drought, salinity, shattering) stresses. It has a thinner seed coat, contributing to its higher percentage of protein and oil content. However, its oil and seed meal contains very high amounts of two major antinutrients—erucic acid and glucosinolates—limiting their utilization in food and animal feed sector [12]. *B. juncea* oil being rich in erucic acid provides very good lubrication and combustion properties, hence is preferred in the paint and automobile industry for biodiesel production [13].

Ensuring the food security of the increasing world population coupled with the continuously decreasing agricultural area due to soil degradation issues such as salinization, is a major challenge for global agricultural production currently [13]. Soil salinity is one of the most significant abiotic factor which seriously hampers the crop production and poses serious threat to world’s food security [14]. According to FAO (2024), 1381 Mha of soils are saline, comprising 20% irrigated and 10% of the cultivated lands globally [15], with an annual increment of 1–2 million ha [16]. The proportion of salt affected lands is projected to render up to 50% of agricultural lands barren and unproductive by the mid 21^st^ century [16–19]. In Pakistan the scenario is even worse with approximately 6.22 million ha of salt affected lands constituting 20.2% of the total arable lands in the country hence threatening national food security and agriculture based industaries [20]. This issue gets critical specially for *Brassica*, primary oilseed crop in Pakistan. With continuously increasing population and improving living standards, Pakistan continues to face the widening gap between domestic edible oil production and consumption relying heavily on costly imports [21,22]. *Brassica* crops are mainly grown on marginal salt affected lands in Pakistan due to its competition with wheat the major staple crop of the country. Moreover, limited availability of high-yielding salt tolerant cultivars poses the major bottleneck in achieiving the sustainable oilseed production. Use of saline lands for *Brassica* crop production results in significant yield reduction and quality deterioration in terms of oil and protein content and fatty acid profile. Addressing this issue is crucial not only for reducing dependence on costly imports but for also promoting agricultural diversification and food security particularly in Punjab region where these crops are well suited for cultivation [23].

Salt stress affects crop productivity by affecting all growth stages with varying levels of effect on each stage and each crop [24]. The effects of salinity can be classified into three broad categories: a) reduction in water potential, b) induction of ion toxicity, and c) nutritional imbalance. Salinity-induced reduction in the leaf water potential in turn reduces turgor pressure and causes osmotic stress [25]. Ion toxicity triggers excess ROS (reactive oxygen species) production, disrupting vital cellular functions and potentially causing DNA and protein damage [26]. Nutritional imbalance coupled with osmotic stress and increased ROS production severely affects plant morphology, biochemistry, and biomass production [27]. In the case of *Brassica* crops, salinity affects the crops at all growth stages from germination to maturity, and overall crop performance [28,29]. Although moderate tolerance to salinity has been observed in some of the *Brassica* species, higher levels of salt above 150–200 mM result in significant yield losses as well as deterioration of quality parameters, for example, increased amounts of glucosinolates and erucic acid in the seed meal and oil, respectively [30–33]. The traits most affected by salinity in *Brassica* crops include plant height, plant dry weight, yield, and product quality [34]. The oil content of the *Brassica* crops was found to be reduced up to 5–7% due to the disproportionate absorption of toxic ions that interfere with the metabolic processes involving seed growth and crop maturity [35,36]. Higher levels of salinity have also been found to reduce the total protein by 15–20% and crude fiber content by 29–34%. However, an upsurge of 12–17% was observed in the erucic acid content under higher salt concentrations, prompting us to investigate the effect of salt stress on genes involved in fatty acid metabolism with a particular focus on euric acid in two widely cultivated species [37]. *B. juncea* is a native to the Asian continent, and therefore shows better tolerance to environmental stresses. It grows better under saline and arid conditions. However, it contains large quantities of erucic acid in its oil, making it unhealthy for human consumption. *B. juncea* cultivars has been shown to increase erucic acid concentrations when exposed to salinity stress [35].

Erucic acid is an omega-9, 22-carbon long monounsaturated fatty acid [38]. It is abundantly present in the oil of *Brassicaceae* crops with maximum levels of up to 50% of the total seed oil in mustard [39]. Erucic acid intake is linked with cardiovascular diseases such as myocardial lipidosis [40]. On the other hand, these very long-chain fatty acids (VLCFAs) together with their derived and conjugated compounds are assumed to be crucial for plant survival, growth, and development [41–43]. Erucic acid and all other VLCFAs are synthesized in the endoplasmic reticulum in a complex process of fatty acyl elongation [44]. The process involves cyclic repetition of four steps—condensation, keto-reduction, dehydration, and enoyl-reduction—regulated by the fatty acid elongase complex, which consists of enzymes specific to each step [45]. Among the enzymes in the fatty acid elongase complex, *Fatty Acid Elongase 1* (*FAE1*)-encoded keto-acyl synthase (KCS) has been identified as key enzyme to erucic acid production in *Brassica* species [46]. In diploid *Brassica* crops such as *B. campestris*, only one *FAE1* gene controls the production of erucic acid but in the case of allotetraploid Brassicas such as *B. napus*, two gene copies of the *FAE1* gene present in each of constituent genome additively involve in the production of erucic acid [47–49]. Another gene *Fatty Acid Desaturase 2* (*FAD2*) plays a crucial role in fatty acid biosynthesis catalyzing the synthesis of linoleic acid from oleic acid, an essential polyunsaturated fatty acid [50]. Studies have demonstrated that simultaneous knockdown of the *FAE1* and *FAD2* genes leads to a reduction in both erucic acid and linoleic acid levels, while significantly increasing oleic acid content [43,51]. Given that salinity stress has been stated to increase erucic acid levels in *Brassica* plants, our study aimed to investigate the impact of NaCl stress on *FAE1* and *FAD2* expressions. These genes have been extensively studied as targets for reducing erucic acid content in *B. juncea* through RNAi and CRISPR technologies. Salt tolerance depends on membrane fluidity and permeability. *FAE1* gene regulates the long chain fatty acids which help in maintaining the structural integrity of the cell. *FAD2* increases unsaturated fatty acids which have role in improvement of membrane flexibility. Since membrane composition directly influences plant tolerance to osmotic and ionic stress, analyzing these genes in *Brassica* provides insights into fatty acid metabolism, molecular adaptation mechanisms under stress conditions and supports the development of salt-tolerant crop varieties.

This study aimed to investigate the role of FAE1 and FAD2-coding genes in *B. juncea* under NaCl-induced salt stress through an integrated molecular, physiological, and agronomic approach. Although FAE1 and FAD2 are key enzymes involved in erucic acid biosynthesis and fatty acid desaturation, respectively, their regulatory behavior and functional contribution under salinity stress remain insufficiently understood. Given that salt stress disrupts membrane integrity, lipid homeostasis, and metabolic balance, genes involved in fatty acid elongation and desaturation may play a critical role in maintaining membrane stability and stress adaptation. We therefore hypothesized that *FAE1* and *FAD2* contribute to salt tolerance by modulating fatty acid composition, thereby influencing membrane functionality, stress signaling, and ultimately agronomic performance.

To test this hypothesis, bioinformatics and promoter analyses were conducted to identify stress-responsive cis-elements and potential regulatory mechanisms. Salt stress was imposed at the seedling stage to evaluate early physiological adaptation, and tissue-specific gene expression analyses were performed to assess spatial regulation under stress conditions. At maturity, yield-related traits were measured to link molecular responses with agronomic performance, while NIR-based seed quality analysis provided biochemical validation of stress-induced changes in lipid composition. This integrated framework establishes a continuum from gene regulation to phenotypic and seed-quality outcomes, offering mechanistic insight into lipid metabolism–mediated stress resilience in *Brassica*.

## Methods

### Bioinformatics analyses

FAE1 and FAD2 protein sequences from *Arabidopsis* were obtained from the TAIR database, and their potential domains were identified using HMMER v3.4 against the Pfam database. These domain sequences were then submitted to BLASTP search in the BRAD database to retrieve *Brassica* protein sequences. Only sequences with an e-value ≤ 1 x 10 ⁻¹⁰, > 90% query coverage, and >80% identity were selected for further bioinformatics analysis.

Key gene features, including chromosome location, gene size, structure, and transcript/protein length, were retrieved from BRAD. The ExPASy tool provided molecular weight, GRAVY values, and isoelectric points. Subcellular localization predictions were made using WoLF PSORT. Protein sequence alignment, similarity, and divergence were assessed via MEGA7, which was also used to construct unrooted neighbor-joining phylogenetic trees with 1,000 bootstrap replicates, and visualized with iTOL. Full-length coding and genomic sequences of *FAE1* and *FAD2* for *Arabidopsis* and *Brassica* species were obtained from BRAD, with gene structures displayed using Gene Structure Display Server 2.0 while conserved domains were verified with NCBI Conserved Domain Database. Protein motifs were identified using MEME (Multiple Em for Motif Elicitation), and cis-acting elements in the 2 kb upstream region were identified with PlantCARE and visualized with TBtools.

### Plant material, growth conditions, and stress treatment

Two *Brassica* species with contrasting erucic acid contents—*B. juncea* cv Super Raya and *B. napus* cv Westar—were used in this study. *B. juncea*, commonly known as Indian mustard or brown mustard, is a widely cultivated *Brassica* species in the Indian subcontinent as well as China [44]. It is better adapted to harsh climatic conditions, shows less pod shattering, and possesses a higher level of resistance against blackleg disease. It contains much higher levels of erucic acid (up to 50% of total seed oil) compared with *B. napus* [45]. *B. napus* contains much lower levels of erucic acid (<2% of the total seed oil) and is a major oilseed crop of the Northern Hemisphere [42]. Seeds of Super raya (*B. juncea*) and Westar (*B. napus*) were kindly provided by the Oilseeds Research Institute, Faisalabad. The experiment was executed in pots lined with polyethen bags to avoid leaching of the salts applied. The seeds of the varieties were sown in a mixture of farmyard manure, soil and sand in 1:1:1 ratio and provided with optimum growth conditions of 25/16 ± 2 °C day/night temperature, 16 h light/8 h dark period in the glass house. The experiment was sown in two sets. One set was used for the data recording of growth parameters after germination while the other set was grown to maturity for data recording of yield parameters, seed biochemical analysis through NIR and qPCR analysis. After successful germination, the plants were thinned to three plants/ pot at uniform growth stage and irrigated with 0, 100, or 200 mM NaCl in 0.5x Hoagland solution. NaCl was given in increments of 50 mM until the required concentration was acheived. Later on the plants were irrigated with simple 0.5x Hoagland solution till the completion of experiment. A randomized complete block design (RCBD) was used with one replication per treatment/block and repeated three times. Samples from siliques, flowers, leaves, stems, and roots of control and treated plants were collected for RT–qPCR, immediately frozen in liquid nitrogen, and stored at −80 °C for RNA extraction.

### Determination of growth parameters

The growth parameters data was taken at 2–week stage after completion of germination. For this purpose, the young plants were uprooted gently from the pot not damaging the roots and washed with distilled water to remove soil from roots. The roots were then separated from the shoot using blade and root and shoot length was measured using standard scale ruler. The roots were kept immersed in the distilled water to avoid dehydration. The root and shoot fresh weight was measured using physical balance. After that, the roots and shoots were oven dried for three days to remove the water content in them. The dry weights were taken using physical balance afterwards.

### Extraction of RNA, cDNA synthesis, and qPCR

RNA extraction of plant tissues was performed manually using tri-reagent from Molecular Research Center lnc. (MRC). Plant samples of about 200 mg were ground into fine powder using liquid nitrogen and immediately transferred to pre-cooled 1.5 ml eppendorf tubes. One ml tri-reagent was added into the powder. The mixture was vortexed to homogenize tri-reagent and powder and kept on ice for 10 min. Other samples were also ground and treated with tri-reagent by the same procedure. Samples were vortexed 2–3 times briefly during this time. After processing, all the samples were kept at room temperature for 5 min. After treating all the samples with tri-reagent, all of them were centrifuged at 12,000 *g* and 4 °C for 10 min. The supernatant containing RNA was collected in new sterile eppendorf tube. 200 µl chloroform was added in the supernatant collected in separate eppendorf tube and mixed with supernatant by vigorously shaking the tubes for 15 sec. The tubes were left for 5 min at room temperature followed by centrifugation at 10,000 *g* and 4 °C for 15 min. Upper aqueous phase was collected in a new sterile eppendorf tube without disturbing the interphase and 300 µl of each of isopropanol and NaCl/Na-citrate salt solution (0.8 M Na-citrate, 1.2 M NaCl) was added. The tubes were inverted to mix all the ingredients and left on the bench for 5–10 min. The tubes were again subjected to centrifugation at 10,000 *g* and 4 °C for 10 min. By the end of this, RNA pellet was obtained at the bottom of the eppendorf tube and supernatant was discarded. Then RNA pellet was washed carefully with 1 ml of 75% ethanol diluted in DEPC-treated water. The tubes were again centrifuged at 10,000 *g* and 4 °C for 10 min and 75% ethanol was removed carefully without losing RNA pellet. The tubes were then left on the bench for 5–10 min opened for air drying. Finally, the pellet was dissolved in 50 µl DEPC treated sterile water and stored at –80 °C.

The quality and integrity of RNA was confirmed using NanoDrop™ 2000/2000c Spectrophotometer (Thermo Scientific Inc.) as well as 1.2% denaturing agarose gel electrophoresis. Residual DNA contamination was removed by treating RNA with RNase-free DNase I (Thermo Sci-entific Inc.) and dilutions were made so that each sample contained an equal amount of RNA. A 5 µg of RNA was used to synthesize cDNA using RevertAid First Strand cDNA Synthesis Kit (Thermo Scientific Inc.).

Complementary DNA (cDNA) was synthesized from RNA samples using the RevertAid First Strand cDNA Synthesis Kit (Thermo Scientific, USA). All the reagents of kit were thawed on ice and centrifuged briefly to collect them at bottom of the tube. Small sterile PCR tubes were taken and labelled. Mixture of 5 µl of total RNA, 7 µl of nucleases free water and 1 µl of oligo (dT)18 were added into the PCR tubes kept in thermal cycler at 65 °C for 5 min to denature RNA. The PCR tubes were then quickly placed on ice. Master mix was prepared in sterile eppendorf tube by adding 24 µl of 5x RT buffer, 6 µl of dNTPs, 6 µl of RNAse inhibitor and 6 µl of M-MuLV RT. Then 7 µl of master mix was added in all the PCR tubes having RNA. The tubes were then spun quickly and incubated at 42 °C for one hour followed by heating at 70 °C for 5 min to inactivate the reaction. In this way 20 µl cDNA was synthesized which was diluted by adding 20 µl of nuclease-free water hence making the total volume of 40 µl. This cDNA was then stored at –80 °C for future use.

Confirmation of successful cDNA synthesis was done by PCR using cDNA as a template and *ACTIN* gene primer. All the reagents of PCR were thawed on ice and quickly spun. The master mix was prepared on ice in a sterile eppendorf tube. The reagents were mixed together and the reaction mixture contained 1x Taq buffer, 2 mM MgCl_2_, 0.2 mM dNTPs, 0.8 µM each forward and reverse primer and 2 U Taq DNA polymerase and 2 µl of cDNA template was added in PCR tube. PCR conditions were: initial denaturation at 94 °C for 1 min, denaturation at 94 °C for 10 sec, primer annealing at 55 °C for 20 sec, extension at 72 °C for 30 sec and final extension at 72 °C for 5 min. The PCR was run for 25 cycles. The cDNA was confirmed by running and visualizing PCR product using agarose gel electrophoresis.

Details of primer sequences are mentioned in S1 Table. Quantitative real-time PCR (qRT–PCR) was conducted using the Bio-Rad CFX96 Touch Real-Time PCR system. qPCR reaction was prepared by adding 6.25 µl of 2x bright green master mix from Applied Biological Material lnc. Canada, 0.25 µl of each of the forward and reverse primer stock (10 µM), 3–4 µl of cDNA template and nuclease-free water to make a total volume of 12.5 µl. PCR conditions followed were 95 °C for 5 min followed by 40 cycles of 95 °C for 30 sec, 55 °C for 30 sec and 72 °C for 30 sec. Melt curve analysis was then performed by increasing temperature from 55 °C to 95 °C and finally keeping 95 °C for 5 sec. Expression of target genes was normalized relative to expression of the *Actin* gene as an internal control. The experiment was performed in triplicates. Relative expression of the target genes was calculated by the 2^-^^∆∆^^CT^ method using the following formulas:

∆CT(test)= CT(target, test) – CT(reference, test)

∆CT(calibrator)= CT(target, calibrator) – CT(reference, calibrator)

∆∆CT = ∆CT(test) – ∆CT(calibrator)

2^-∆∆CT^ = Normalized expression ratio

### Determination of yield related traits

Plants were harvested for the evaluation of yield parameters after seed formation in the pods but before pod shattering began to prevent seed loss. Prior to harvesting, plant height and the number of primary branches were recorded at full maturity. Afterward, all siliques from each plant were removed, collected in separate paper bags, and counted. Silique length and the number of seeds per silique were measured using ten randomly selected siliques from each plant. Finally, all seeds from each plant were collected and weighed to determine the seed yield per plant.

### Estimation of seed quality parameters

The seed oil, protein, and fatty acid profiles were assessed using a non-invasive near-infrared reflectance spectroscopy (NIR) method. A DA 7250 At-Line NIR analyzer, calibrated with oilseeds of known concentrations of oil, protein, and fatty acids, was employed for this analysis. A partial least squares regression approach was utilized to establish a statistical relationship between the spectral data and the concentrations of the respective components.

### Statistical analyses

Statistical analyses were conducted using R (version 4.1.3) for the Windows. One-way or two-way ANOVA, as appropriate, were performed to evaluate the effects of NaCl treatment and genotype on plant growth attributes, gene expression and seed quality parameters, including oil, protein, and fatty acid composition. Differences among means were further analyzed using Tukey’s Honestly Significant Difference (HSD) test with a significance level set at α = 0.05.

## Results

### Identification of *FAE1* and *FAD2* genes in *Brassica* complex

The protein sequences for *FAE1* and *FAD2* genes were obtained from all six *Brassica* species using their respective potential domains from Arabidopsis *FAE1* and *FAD2* as queries. A total of 9 and 11 putative protein sequences from *Brassica* species were identified for FAE1 and FAD2 respectively. Diploid species contained single whereas allotetraploid species comprised two copies of the respective genes except for *B. oleracea* and *B. carinata* which attained 2 and 3 *FAD2* gene copies, respectively. The amino acid length, protein weight, GRAVY values, and pI for all the FAE1 putative proteins ranged from 475 aa to 968 aa, 52.9 kDa to 108.7 kDa, 9.26 to 9.39 and −0.129 to −0.052, respectively. The amino acid length, protein weight, GRAVY values, and pI of *Brassica* FAD2 proteins ranged from 384 aa to 469 aa, 43.9 kDa to 54.1 kDa, 8.25 to 9.21, and –0.309 to –0.121, respectively. All of the FAE1 proteins were predicted to be located on the plasma membrane while all the FAD2 proteins were predicted to be found on endoplasmic reticulum. However, four of them (BolFAD2.1, BraFAD2, BnaFAD2.1, and BjuFAD2.2) were also found to be located on plasma membrane (S2 Table).

### Phylogenetic analysis of FAE1 and FAD2 proteins

**Fig 1:**
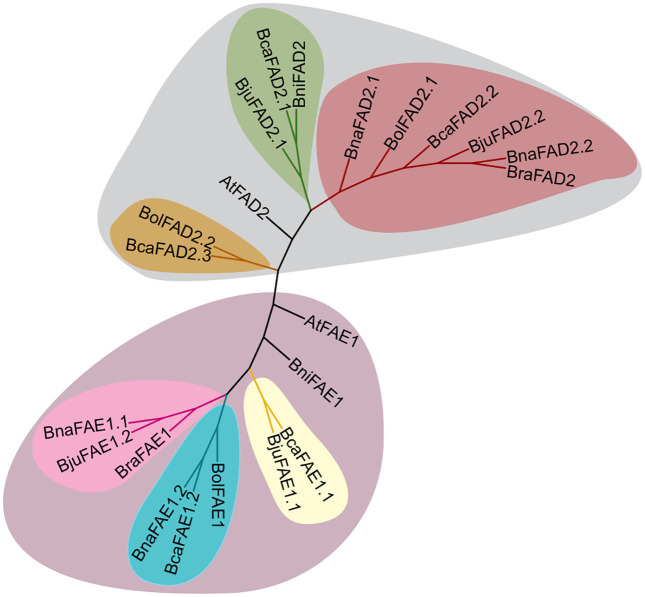
Phylogenetic tree of FAE1 and FAD2 proteins from Arabidopsis and six *Brassica* species of U’s triangle (*B. nigra*, *B. oleracea*, *B. rapa*, *B. carinata*, *B. juncea* and *B. napus*). Different phylogenetic groups are represented in different colors. The accession numbers for query proteins are given as follows: AtFAE1 (AT4G34520), BolFAE1 (BolC3t20858H), BniFAE1 (BniB03g020700.2N), BraFAE1 (BraA08g016330.3.5C), BnaFAE1.1 (A08p16760.1_BnaDAR), BnaFAE1.2 (C03p85720.1_BnaDAR), BjuFAE1.1 (BjuVB03G19720), BjuFAE1.2 (BjuVA08G16890), BcaFAE1.1 (BcaB03g11828), BcaFAE1.2 (BcaC01g00899), AtFAD2 (AT3G12120.1), BolFAD2.1 (BolC5t34237H), BolFAD2.2 (BolC1t05264H), BniFAD2 (BniB01g052830.2N), BraFAD2 (BraA05g035880.3.5C), BnaFAD2.1 (C05p55690.1_BnaDAR), BnaFAD2.2 (A05p37230.1_BnaDAR), BjuFAD2.1 (BjuVB01G42140), BjuFAD2.2 (BjuVA05G37350), BcaFAD2.1 (BcaB06g25543), BcaFAD2.2 (BcaC05g29288) and BcaFAD2.3 (BcaC09g48213).

A phylogenetic tree constructed using FAE1 and FAD2 protein sequences from *Brassica* and Arabidopsis. The analysis revealed distinct divergence patterns for FAE1 and FAD2 proteins. For FAE1 proteins, the tree exhibited three distinct branches corresponding to their respective sub-genomes, with each branch containing proteins from the same sub-genome. In particular, sister proteins from the allotetraploid species show greater similarity to each other compared to their diploid parent proteins. The FAE1 protein from *B. nigra* diverged distinctly from all other proteins. FAD2 proteins from the A and C genomes clustered together, while proteins from the B genome formed a separate cluster. Notably, additional copies of FAD2 proteins in *B. oleracea* and *B. carinata* diverged distinctly from the other FAD2 proteins, highlighting their unique evolutionary paths. Additionally, FAE1 and FAD2 proteins from Arabidopsis were positioned separately from their *Brassica* counterparts, reflecting evolutionary divergence from their ancestral forms in Arabidopsis (Fig 1).

A multiple sequence alignment of FAE1 and FAD2 proteins from six *Brassica* species and Arabidopsis was performed using the MUSCLE to investigate amino acid-level similarities and differences. The alignment revealed distinct differences between FAE1 and FAD2 proteins; however, there was a high degree of similarity among proteins within the same clade. The overall similarity percentage among all FAE1 and FAD2 proteins ranged from 17% to 100%. Specifically, within the FAE1 group, similarity percentages ranged from 85% to 99%, and within the FAD2 group, they ranged from 85% to 100% (Table 1). Correspondingly, divergence percentages calculated from pairwise distances were higher for proteins with lower similarity percentages and lower for those with higher similarity percentages.

**Table 1:** Pairwise sequence resemblance and divergence percentages between FAE1 and FAD2 proteins.

Similarity (%)																							
		AtFAD2	BolFAD2.1	BolFAD2.2	BniFAD2	BraFAD2	BnaFAD2.1	BnaFAD2.2	BjuFAD2.1	BjuFAD2.2	BcaFAD2.1	BcaFAD2.2	BcaFAD2.3	AtFAE1	BolFAE1	BniFAE1	BraFAE1	BnaFAE1.1	BnaFAE1.2	BjuFAE1.1	BjuFAE1.2	BcaFAE1.1	BcaFAE1.2
**Divergence (%)**	AtFAD2		90.6	85.1	90.9	90.3	90.6	90.3	90.3	90.3	91.4	90.6	85.1	17.9	17.9	19.0	17.9	17.9	18.3	18.2	17.9	18.2	17.7
	BolFAD2.1	9.1		90.6	96.1	98.0	99.6	98.0	96.6	98.3	96.6	100.0	90.6	18.4	18.7	18.3	18.7	18.7	18.6	18.9	18.7	18.9	18.4
	BolFAD2.2	15.9	10.0		89.3	90.6	90.6	90.6	89.6	90.9	89.6	90.6	98.7	18.7	18.9	20.0	18.9	18.9	19.2	19.2	18.9	19.2	18.7
	BniFAD2	8.9	3.1	11.3		95.8	96.1	95.8	99.5	96.1	99.5	96.1	89.8	18.4	18.7	20.0	18.7	18.7	18.9	18.9	18.7	18.9	18.4
	BraFAD2	9.8	0.6	10.4	3.8		98.0	100.0	96.4	99.8	96.4	99.2	90.6	17.8	18.1	17.8	18.1	18.1	18.3	18.4	18.1	18.4	17.8
	BnaFAD2.1	9.4	0.3	10.3	3.5	0.9		98.0	96.6	98.3	96.6	99.5	90.6	18.4	18.7	18.3	18.7	18.7	18.9	18.9	18.7	18.9	18.4
	BnaFAD2.2	9.8	0.6	10.4	3.8	0.0	0.9		96.4	99.8	96.4	99.2	90.6	17.8	18.1	17.6	18.1	18.1	18.3	18.4	18.1	18.4	17.8
	BjuFAD2.1	8.5	2.8	11.0	0.3	3.5	3.2	3.5		96.6	100.0	96.6	90.1	18.4	18.7	20.0	18.7	18.7	18.9	18.9	18.7	18.9	18.4
	BjuFAD2.2	9.5	0.3	10.1	3.5	0.3	0.6	0.3	3.2		96.6	99.5	90.9	17.8	18.1	17.8	18.1	18.1	18.3	18.4	18.1	18.4	17.8
	BcaFAD2.1	8.5	2.8	11.0	0.3	3.5	3.2	3.5	0.0	3.2		96.6	90.1	18.4	18.7	20.0	18.7	18.7	18.9	18.9	18.7	18.9	18.4
	BcaFAD2.2	9.1	0.0	10.0	3.1	0.6	0.3	0.6	2.8	0.3	2.8		90.6	18.4	18.7	18.3	18.7	18.7	18.6	18.9	18.7	18.9	18.4
	BcaFAD2.3	16.1	10.1	1.6	10.8	10.5	10.4	10.5	10.4	10.2	10.4	10.1		18.4	18.9	20.0	18.9	18.9	19.2	19.2	18.9	19.2	18.7
	AtFAE1	260.3	253.4	246.4	245.5	255.3	251.9	255.3	246.6	254.7	246.6	253.4	248.3		85.5	85.0	85.7	85.9	85.7	86.5	85.9	86.7	85.4
	BolFAE1	250.2	243.9	237.8	236.8	245.7	242.6	245.7	237.9	245.2	237.9	243.9	237.5	16.0		96.1	99.0	98.8	99.8	97.4	99.2	97.2	99.6
	BniFAE1	247.7	241.0	234.9	234.0	242.7	239.7	242.7	235.0	242.3	235.0	241.0	234.6	16.8	4.6		95.7	95.5	96.2	98.4	95.9	98.2	95.7
	BraFAE1	250.4	244.1	237.9	236.9	246.3	242.7	246.3	238.0	245.4	238.0	244.1	237.6	16.0	1.2	4.9		99.4	98.7	97.2	99.8	97.0	98.6
	BnaFAE1.1	251.2	244.9	238.7	237.7	246.6	243.5	246.6	238.7	246.2	238.7	244.9	238.2	15.6	0.9	4.6	0.3		98.5	97.0	99.6	96.8	98.4
	BnaFAE1.2	250.2	243.9	237.8	236.8	245.7	242.6	245.7	237.9	245.2	237.9	243.9	237.5	16.0	0.0	4.6	1.2	0.9		97.5	99.0	97.3	99.4
	BjuFAE1.1	253.1	246.7	240.4	239.3	248.5	245.3	248.5	240.4	248.0	240.4	246.7	240.0	14.3	2.7	2.1	2.7	2.4	2.7		97.4	99.8	97.0
	BjuFAE1.2	251.2	244.9	238.7	237.7	246.6	243.5	246.6	238.7	246.2	238.7	244.9	238.3	15.6	0.9	4.6	0.3	0.0	0.9	2.4		97.2	98.8
	BcaFAE1.1	253.8	247.0	240.7	239.7	248.8	245.7	248.8	240.7	248.3	240.7	247.0	240.4	13.9	3.0	2.4	3.0	2.7	3.0	0.3	2.7		96.8
	BcaFAE1.2	253.4	247.1	240.8	239.8	248.9	245.7	248.9	240.8	248.4	240.8	247.1	240.5	16.3	0.6	5.2	1.8	1.5	0.6	3.3	1.5	3.6	

Values highlighted in blue and gray denote similarity and divergence, respectively. The intensity of the colour corresponds to the magnitude of the value.

### Gene structure, domain, and motif analyses of FAE1 and FAD2 proteins

**Fig 2:**
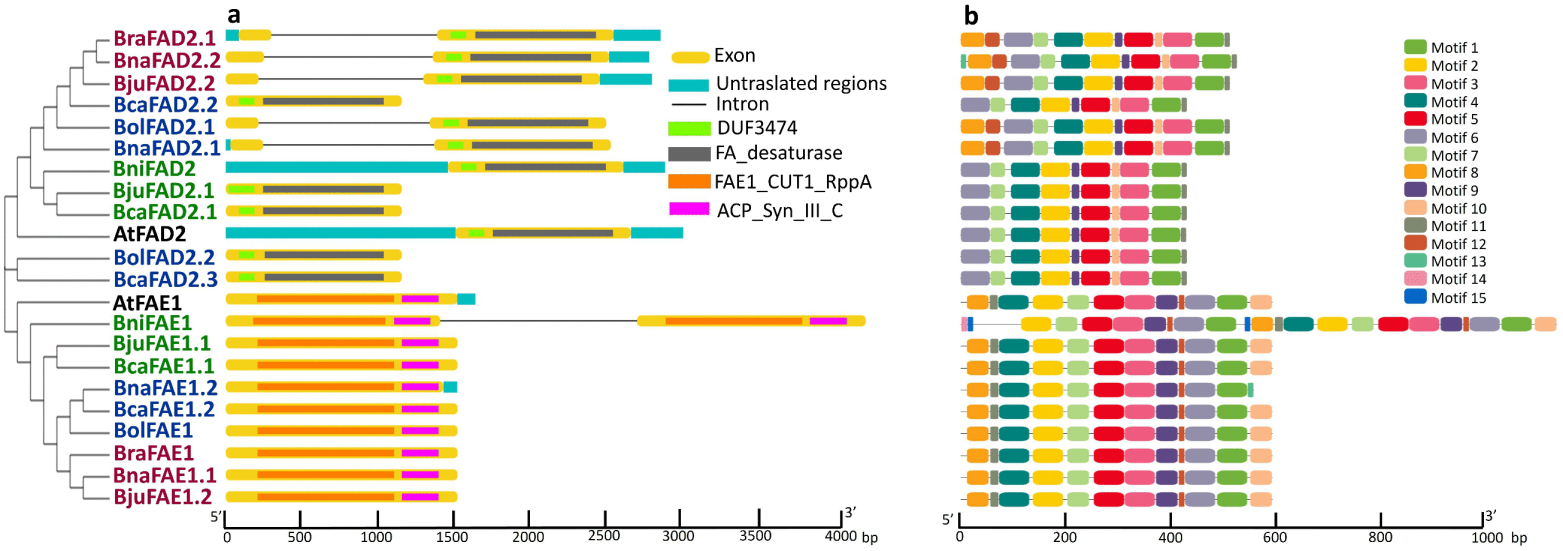
Gene structure, domain and motif analysis of FAE1 and FAD2 proteins from six *Brassica* species and Arabidopsis. (a) Gene structure of FAE1 and FAD2 genes and domain positions were drawn in Gene Structure Display Server (GSDS) by submitting the full length and coding DNA sequences of FAE1 and FAD2 genes and the BED file for the location of domains selecting proteins’ ordinates. The colored rectangles represent different gene and protein features (see legend for details). (b) Motif analysis was performed using MEME suite v5.5.5 and a total of 15 and 13 statistically significant motifs were identified among the query proteins of FAE1 and FAD2, respectively, which are represented by different colors as given in the legend. See caption of Fig 1 for the accession numbers of the proteins. Motif sequences found in FAE1 and FAD2 proteins are given in S3 Table.

Gene structures of *FAE1* and *FAD2* were deduced by employing GSDS online resources. *FAE1* genes showed higher levels of conservation in terms of retaining exons as each of *FAE1* genes consisted of a single exon just like *AtFAE1* except *BniFAE1* which comprised two exons of comparable lengths. Additionally, most of the *FAE1* genes lost their 5′ UTRs which were found to be present in *AtFAE1*. Six out of 10 *FAD2* genes in *Brassica* species comprised of only a single exon similar to *AtFAD2* gene. However, five of the remaining genes comprised of one additional and comparatively smaller exon besides the other common and larger exon found in all other genes. *FAD2* genes bearing two exons also attained a large intron hence increasing the gene size. Only one gene possessed 3′ and 5′ UTRs similar to the *FAD2* gene in Arabidopsis while most of the genes lost their UTRs in the course of evolution (Fig 2a).

FAE1 and FAD2 proteins were examined for the presence of putative domains by submitting the protein sequences to the NCBI CD database and further selecting the Pfam v340-19178 PSSMs database. All the FAE1 proteins from *Brassica* species comprised of FAE1_CUT1_RppA and ACP_Syn_III_C domains crucial for the activity of FAE1 proteins just like their parent protein from Arabidopsis (AtFAE1). Domain analysis of FAD2 proteins confirmed that all the proteins in *Brassica* species comprised of putative FA desaturase superfamily and DUF3474 superfamily domains crucial for the function of the FAD2 proteins similar to AtFAD2 protein. A high degree of conservation was observed for having domains in both the FAE1 and FAD2 proteins (Fig 2a).

Motif analysis of FAE1 and FAD2 proteins was performed by submitting the protein sequences to MEME suite v5.5.5 and visualized by TBtools. Fifteen [15] and thirteen [13] putative motifs were found in the FAE1 and FAD2 proteins respectively. FAE1 proteins showed high levels of conservation for the presence of all motifs in each of the member proteins in each species except for little variations. Motif 1 and motif 12 were found to be constitutively present in all FAE1 proteins. BniFAE1 comprised two copies of each of the motifs except motifs 8, 4, 10, and 11. Motif 13 was found to be exclusively present in BnaFAE1.2. Motifs 14 and 15 were only present in BniFAE1 in single and double copies respectively. Motif analysis of FAD2 proteins revealed that most of the motifs were constitutively present in all FAD2 proteins except Motif 8 and 12 which were present in BraFAD2, BnaFAD2.2, BjuFAD2.2, BolFAD2.1 and BnaFAD2.1 only. Motif 13 was found to be exclusively present in BnaFAD2.2 (Fig 2b).

### Analysis of Cis-acting Elements in *FAE1* and *FAD2* Genes

**Fig 3:**
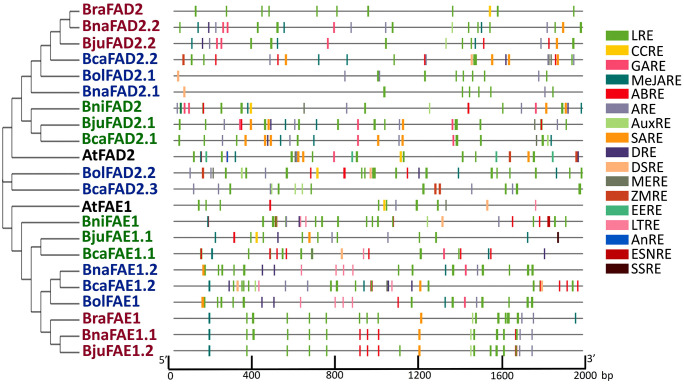
Cis-acting element analysis of the promoter regions of the FAE1 and FAD2 genes from six *Brassica* species and Arabidopsis. Different cis-regulatory elements are represented in different colours as per legend. The cis-regulatory elements are abbreviated as follows: AuxRE, auxin-related responsive element; LRE, light-responsive element; LTRE, low-temperature related element; EERE, endosperm expression regulatory element; ARE, anaerobic induction element; MERE, meristem specific regulatory element; ZMRE, zein metabolism regulatory element; DRE, drought inducibility element; ABRE, abscisic acid-responsive element; MeJARE, methyl jasmonate-responsive element; GARE, gibberellic acid-responsive element; DSRE, defense- and stress-related element; CCRE, circadian control regulatory element; SARE, salicylic acid-responsive element; AnRE, anoxic specific inducibility element; ESNRE, Endosperm specific negative expression element; SSRE, Seed specific regulatory element.

A 2–kb upstream regions of the FAE1 and FAD2 genes were examined to identify cis-regulatory elements utilizing the PlantCARE database. The promoter region analysis resulted in the identification of multiple copies of light-responsive elements (LRE) and anaerobic induction element (ARE) in all gene copies of *FAE1*and *FAD2* genes conferring the significant role of these genes against light stimulus and as well as plant survival under anaerobic conditions. Moreover, hormone-related cis-regulatory elements (ABRE, MeJARE, SARE, GARE, AuxRE) were also widely distributed among most of the gene copies of *FAE1* and *FAD2* genes deducing the role of these genes in hormone regulation mechanisms. Out of all these hormone-related elements, ABRE and MeJARE elements were mostly abundant and constitutively present as compared to SARE, GARE, and AuxRE, which were present in some of the genes and absent in others. Among the stress-related cis-elements, drought inducibility elements (DRE) were also found in most of the gene copies of *FAE1* and *FAD2* genes while defence and stress-related elements (DSRE) and low-temperature regulatory elements (LTRE) were unique to some genes only. Related to tissue-specific expression, meristem expression-related elements (MERE) were abundantly present in the majority of the genes while endosperm expression regulatory element (EERE), Endosperm negative expression related element (ESNRE), and seed-specific related elements (SSRE) were exclusively present in the promoter regions of *BniFAE1*, *BjuFAE1.1* and *AtFAD2* gene respectively. Different other regulatory elements were also identified in *FAE1* and *FAD2* genes such as zein metabolism-related elements (ZMRE), anoxic specific inducibility (AnRE), circadian clock-related elements (CCRE), and cell division related elements (CDRE) conferring the role of these genes in a variety of plant metabolic reactions, growth, and development as well as stress tolerance mechanisms (Fig 3).

### Effect of NaCl stress on growth parameters

**Fig 4:**
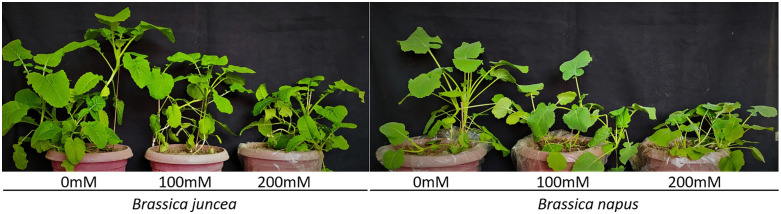
Phenotypic response of *B. juncea* and *B. napus* under control, 100 and 200 mM NaCl stress.

**Fig 5:**
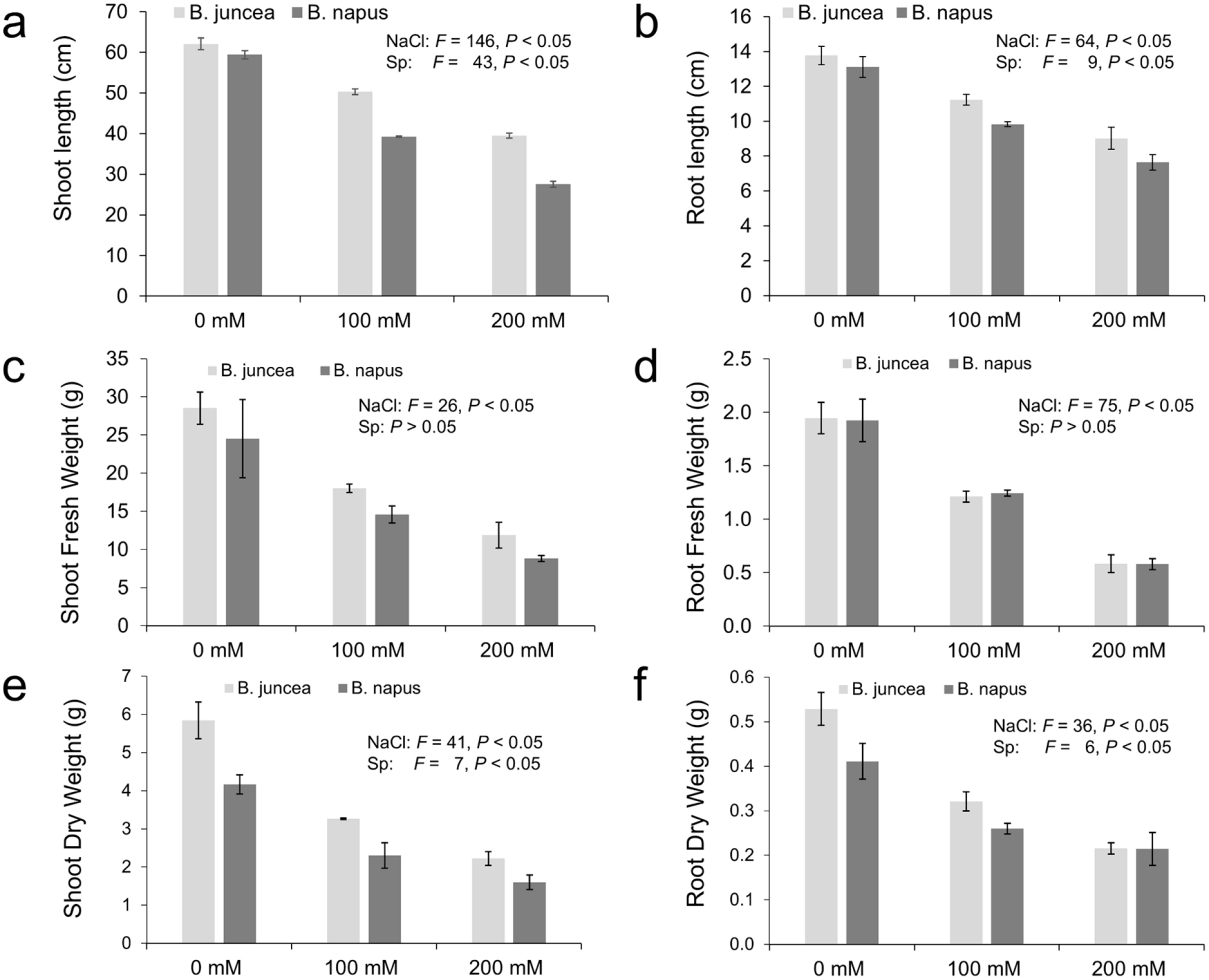
Effect of NaCl stress on the growth attributes in *B. juncea* and *B. napus*. Panel (a-f) represent growth attributes, i.e., shoot length (a), root length (b), shoot fresh weight (c), root fresh weight (d), shoot dry weight (e) and root dry weight (f) in *B. juncea* and *B. napus*, respectively. Gray and black colored bars represent *B. juncea* and *B. napus*, respectively. Each data point displays the mean ± SD calculated from three biological repeats (Tukey’s HSD, *P* < 0.05).

Exposure to salt stress severely reduced all the growth parameters in both the cultivars (Fig 4). The magnitude of reduction increased with increasing NaCl concentration, and was generally higher in *B. napus* as compared to *B. juncea* (Fig 5). For instance, *B. napus* showed ~15% and ~30% more reduction in the shoot length at 100 mM and 200 mM salt concentration respectively compared to *B. juncea* (Fig 5a). Similarly the reduction in root length was ~ 7% higher in *B. napus* as compared to *B. juncea* at both salinity levels. Reductions in root fresh weight and shoot dry weight were comparable between the two species, whereas *B. juncea* exhibited a relatively higher reduction in root dry weight under salt stress (Fig 5f). All treatments showed significant differences (*P* < 0.05).

### Effect of NaCl stress on *FAE1* and *FAD2* genes in different tissues

**Fig 6:**
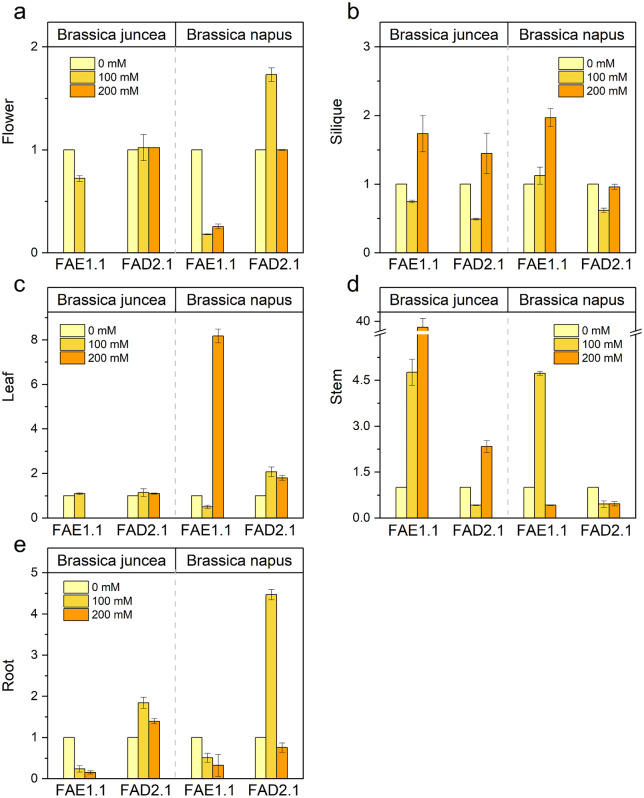
The expression of FAE1.1 and FAD2.1 genes in different tissues of *B. juncea* and *B. napus* under NaCl stress. (a–e) represent gene expression in flower, silique, leaf, stem and root tissue, respectively under control and NaCl stress conditions. Light yellow, yellow and orange colored bars represent 0, 100 and 200 mM NaCl concentrations, respectively. Each data point displays the mean ± SD calculated from three biological repeats (Tukey’s HSD, *P* < 0.05).

Salt stress severely affects membrane stability, ion homeostasis, and overall plant productivity in *Brassica*. Since membrane integrity largely depends on fatty acid composition, genes involved in fatty acid biosynthesis play a crucial role in stress adaptation. *FAE1* regulates the elongation of fatty acids to produce very-long-chain fatty acids important for seed oil composition, while *FAD2* catalyzes the desaturation of oleic acid to linoleic acid, enhancing membrane fluidity under stress conditions. Because salt stress responses are tissue-specific, analyzing the expression of *FAE1* and *FAD2* in different tissues provides insight into spatial regulation of lipid metabolism and its contribution to salt tolerance and oil quality in *Brassica*. The study was planned to explore the effect of NaCl on *FAE1* and *FAD2* genes’ expression patterns involved in the erucic acid biosynthesis process. Given the high similarity between the homeologs of *FAE1* and *FAD2* (99%)*,* expression analysis was conducted on only one homeolog from each gene—*FAE1.1* and *FAD2.1*. The expression of *FAE1.1* was highly induced in silique at 200 mM and in stem at both 100 mM and 200 mM stress, respectively. The expression declined or remained at control levels in the rest of the plant tissues at both salinity levels in *B. juncea* (Fig 6). The *FAE1.1*’s expression was induced in the stem at 100 mM and leaf and silique at 200 mM NaCl levels while exhibiting reduction in the rest of the plant parts for both 100 and 200 mM salinity levels in *B. napus*. The expression of the *FAD2.1* gene was upregulated in roots, and downregulated in siliques and stems while remaining unchanged in other parts (leaf and flower) of *B. juncea* at 100 mM stress. However, as the NaCl concentration reached 200 mM, the expression was highly induced in the root stem and silique. Mild NaCl stress strongly induced the expression of *FAD2.1* in root (5x) and flower (2x) in *B. napus* while remaining unchanged at 200 mM NaCl stress. The gene expression declined or remained unchanged in the rest of the plant parts of *B. napus* at both salinity levels.

### Effect of NaCl stress on yield related traits

**Fig 7:**
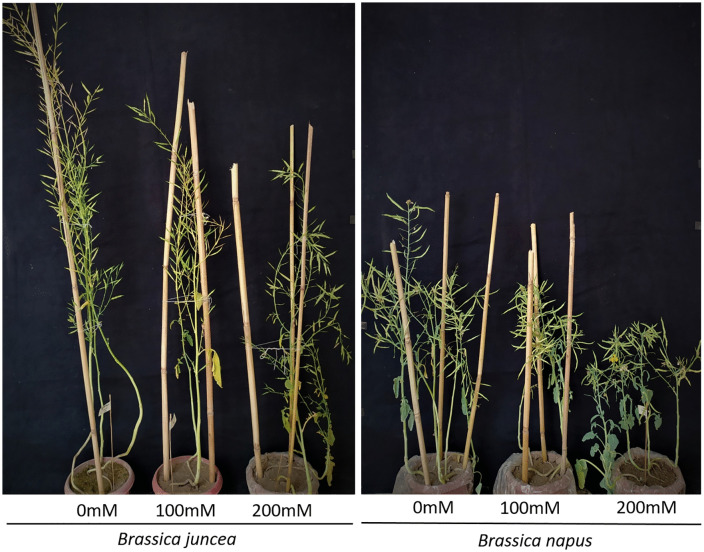
Effect of salt stress on yield parameters of *B. juncea* and *B. napus*. Pictures of representative plants at maturity under 0, 100 and 200 mM NaCl stress.

The yield of both the Brassicas was negatively affected due to NaCl stress as shown by different yield-related parameters like plant height, number of primary branches, number of siliques per plant, silique length, number of seeds per silique and total seed yield per plant (Fig 7). The height of mature plants was negatively affected in both of the species but however the percentage reduction (7% and 20% at 100 mM and 200 mM salt stress, respectively) was comparable in both the species. The number of primary branches was also reduced in both the cultivars, but the reduction was more evident in *B. napus* (31% and 46%) as compared to *B. juncea* (21% and 29%) at 100 mM and 200 mM NaCl stress.

The number of siliques per plant affected in both of the species but the percentage reduction was more in *B. napus* 22% and 35% at 100 mM and 200 mM salt stress compared to moc treated plants. Silique length was also affected in both the Brassicas under the NaCl stress. However, the reduction in silique length was ~ 12% and 9% higher in *B. napus* than *B. juncea* at 100 mM and 200 mM salt stress conditions.

**Fig 8:**
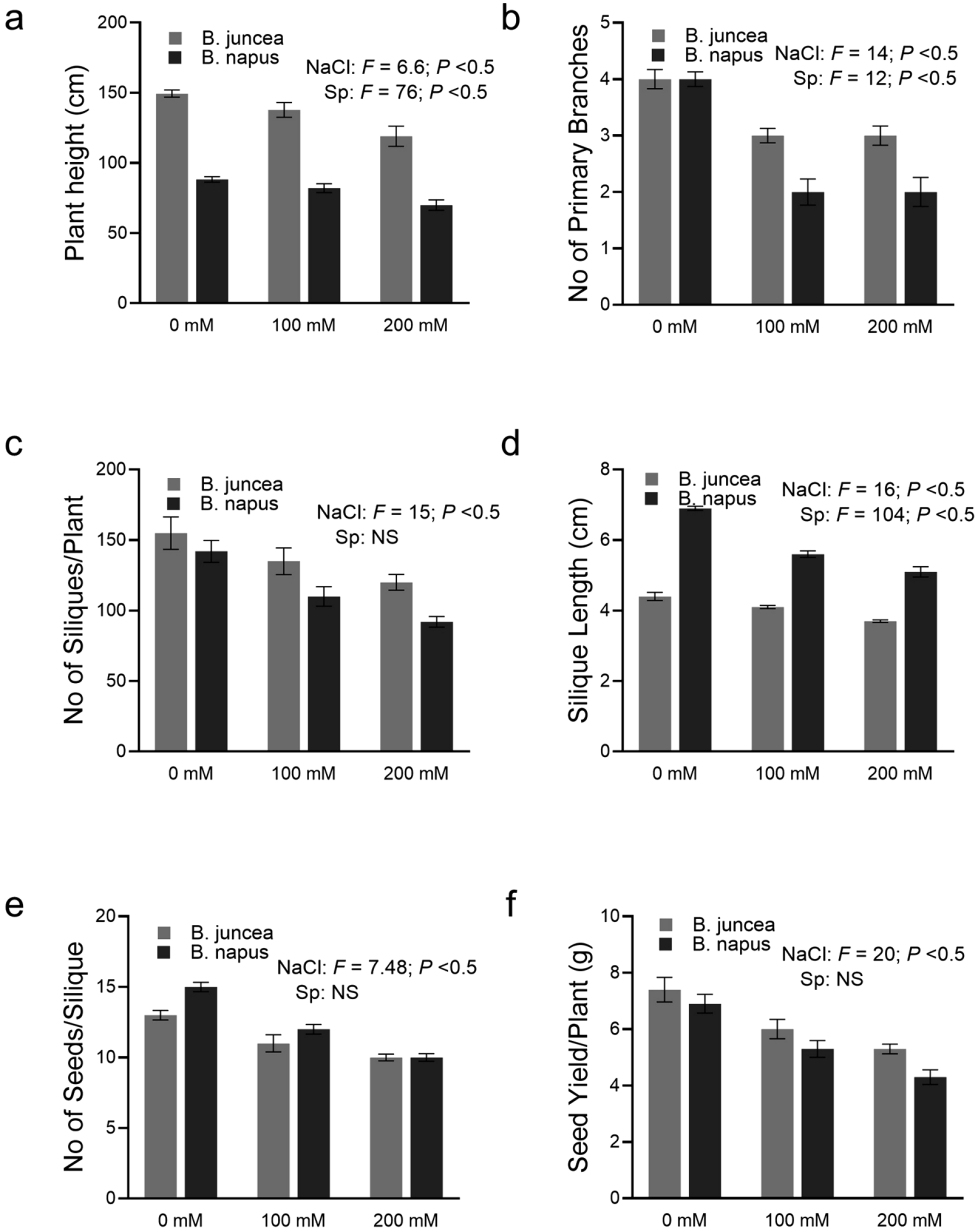
Effect of salinity on growth and yield-related traits of *B. juncea* and *B. napus.* (a) shows plant height, (b) number of primary branches, (c) number of siliques per plant, (d) silique length, (e) number of seeds per silique, and (f) seed yield per plant. Gray and black colored bars represent *B. juncea* and *B. napus*, respectively. Data are presented as mean ± SE of biological replicates (Tukey’s HSD, *P* < 0.05).

Salt stress also affected the number of grains in siliques both of the Brassicas yet the effect was more severe in *B. napus* with 22% and 35% reduction compared with the mock treated plants. Seed yield in both the Brassicas was also recorded to see the extent of the impact of NaCl stress. Both the Brassicas experienced a statistically significant yield decrease, however, once again, this yield reduction was much higher in *B. napus* (23% and 37%) than *B. juncea* (18% and 22%) at 100 and 200 mM salt stress respectively (Fig 8).

### Effect of NaCl stress on oil and total proteins contents

**Fig 9:**
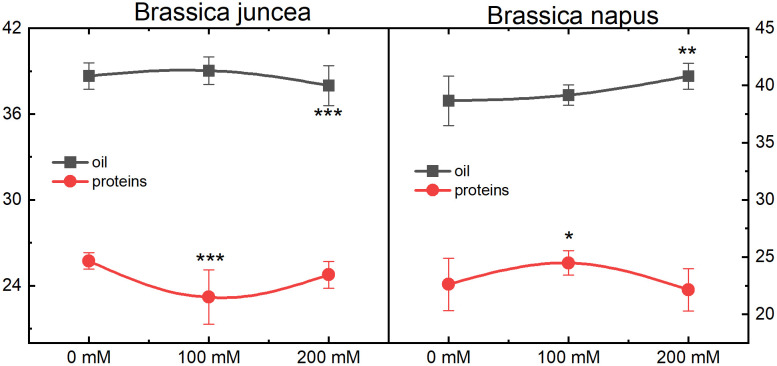
Effect of NaCl stress on the total oil and protein content in the seeds of *B. juncea* and *B. napus.* Black and red lines represent oil and protein content, respectively. Each data point displays the mean ± SD calculated from three biological repeats (Tukey’s HSD, *P* < 0.05).

The effect of varying NaCl concentrations (0, 100, and 200 mM) was determined on oil and total protein percentage in *B. juncea* and *B. napus*. Contrasting trends were observed for oil and protein levels in *B. juncea* and *B. napus*. For oil content, a slight rise was observed in *B. juncea* and *B. napus* at 100 mM NaCl stress. However, both species exhibited contrasting trends, i.e., a decrease in *B. juncea* and an increase in *B. napus* at 200 mM NaCl level. Although, these changes were minute but were statistically significant. This stability suggests that oil production in both species is resilient to NaCl stress. The protein content was decreased in *B. juncea* at 100 mM NaCl stress followed by an increase at 200 mM NaCl stress. The opposite pattern was observed in *B. napus* for protein content, as it increased at 100 mM followed by a decline up to control levels at 200 mM (Fig 9).

Overall, the results indicate that oil and protein content in *B. juncea* and *B. napus* are slightly affected by salinity levels up to 200 mM NaCl. This resilience could be a key element in the ability of these species to tolerate saline environments without compromising their essential biochemical properties.

### Effect of NaCl stress on fatty acid composition

The overall erucic acid content varied significantly between the species (*P* ≤ 2.0 × 10^-16^), underscoring the inherent genetic differences between the *B. juncea* and *B. napus* groups. This variation highlights the distinct metabolic pathways and genetic makeup that contribute to the fatty acid profile of these species.

**Fig 10:**
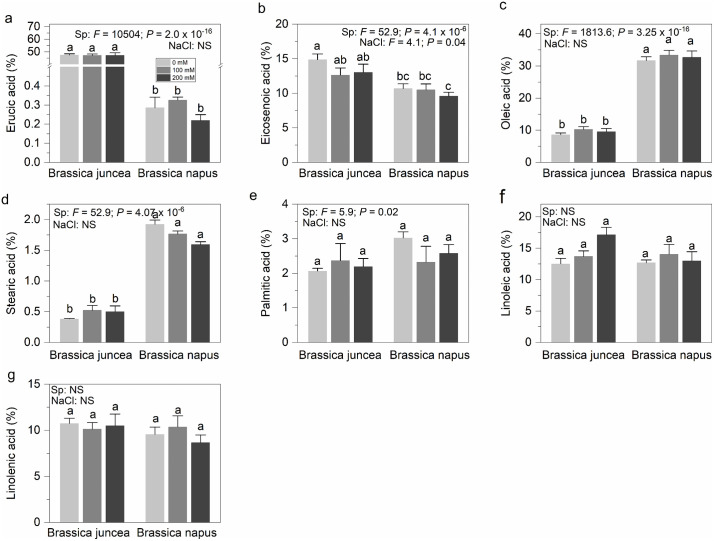
NaCl-induced change in the fatty acid composition in the seeds of *B. juncea* and *B. napus.* (a–g) represent Erucic acid, Eicosenoic acid, Oleic acid, Stearic acid, Palmitic acid, Linoleic acid and Linolenic acid content, repectively. Light gray, gray and black colored bars represent 0, 100 and 200 mM NaCl concentrations, respectively. Each data point displays the mean ± SD calculated from three biological repeats (Tukey’s HSD, *P* < 0.05).

Regarding the influence of NaCl on erucic acid levels, *B. juncea* exhibited stability across all salinity treatments, with no significant changes observed. In contrast, *B. napus* exhibited an initial increase in erucic acid level at 100 mM NaCl, followed by a noticeable decrease at 200 mM NaCl. Despite these fluctuations, the changes were statistically insignificant (*P* > 0.05), suggesting that erucic acid levels in *B. napus* are somewhat resilient to salinity stress (Fig 10a).

A slight but statistically significant reduction (*P* ≤ 0.04) in eicosenoic acid content was observed in both species as NaCl levels increased (Fig 10b). This reduction may indicate that eicosenoic acid is more susceptible to salinity-induced metabolic shifts, which could have implications for the overall fatty acid composition and stability of cellular membranes under stress conditions.

Oleic acid content showed highly significant differences between the two species (*P* ≤ 3.2 × 10^-16^), yet NaCl had no significant impact on its levels (*P* > 0.05) (Fig 10c). This outcome suggests that species-specific factors display a more dominant role in determining oleic acid content than environmental salinity, potentially reflecting underlying genetic control mechanisms that govern oleic acid biosynthesis.

Saturated fatty acids (SFAs), including stearic and palmitic acid, responded differently to increasing NaCl levels (Fig 10d-10e). In *B. juncea*, the content of these SFAs generally increased with higher salinity, possibly as a protective mechanism to enhance membrane rigidity and stability under osmotic stress. Conversely, *B. napus* displayed a decreasing trend in these fatty acids, which might indicate a less effective or different adaptive response to salt stress.

Finally, the impact of NaCl on linoleic acid (essential fatty acid) was statistically insignificant in both species (*P* > 0.05) (Fig 10f). The stability of linoleic acid levels under varying salinity conditions suggests that its biosynthesis is tightly regulated, ensuring that essential physiological functions dependent on this fatty acid are maintained despite environmental stressors.

## Discussion

*Brassica* crops are widely cultivated worldwide for their high-quality vegetable oil, which is recognized for its superior nutritional value. However, the significant presence of erucic acid—comprising up to 50% of the oil in most *Brassica* seeds, except for Canola cultivars—poses health risks, limiting its suitability for human consumption. Salt stress presents a substantial challenge to *Brassica* cultivation, affecting all growth stages and leading to reduced yield, diminished oil content, and increased erucic acid levels [52,53]. Salt stress disrupts membrance integrity and cellular homeostasis. Fatty acid composition determines the membrane fluidity and stress tolerance. The evaluation of *Brassica* species thriving better under saline conditions is crucial in terms of better oil, protein content and better fatty acid profile with reduced erucic acid in order to grow on saline soils. In this way, these saline soils will also be useful for agriculture and it will also add up to the agricultural production and ensuring food security hence contributing to the SDGs goal of zero hunger. The *FAE1* gene plays a critical role in erucic acid biosynthesis, with the *FAD2* gene also influencing its production. This study integrated bioinformatics, gene expression profiling, physiological evaluation, yield analysis, and NIR-based seed quality assessment to investigate the role of *FAE1* and *FAD2* in *Brassica* under salt stress. The findings establish a continuum from molecular characterization and tissue-specific regulation to agronomic performance and oil quality, demonstrating the importance of fatty acid metabolism in salinity tolerance.

Bioinformatics analysis of the *FAE1* and *FAD2* genes, along with their respective proteins, across Arabidopsis and six *Brassica* species provided valuable insights into their phylogenetic relationships, gene structure, domain analysis, motif conservation, and the regulatory elements present in their promoter regions. For example, the identification and characterization of *FAE1* and *FAD2* genes in the *Brassica* complex highlight the evolutionary dynamics of these key genes responsible for fatty acid biosynthesis. BLAST analysis indicated that diploid *Brassica* species preserved a single ortholog for each of the AtFAE1 and AtFAD2 proteins, despite the genome triplication events that occurred approximately 9–15 million years ago within the *Brassicaceae* family [54,55]. In contrast, allotetraploid species exhibited two orthologous proteins for each of the AtFAE1 and AtFAD2 proteins, suggesting that these allotetraploids retained one gene copy from each parent during allopolyploidization [56]. Notably, the extra copies of *FAD2* in *B. oleracea* and *B. carinata*, which diverged distinctly in the phylogenetic analysis (Fig 1), may indicate species-specific adaptations or functional divergence of these gene copies as a result of segmental duplications [57,58]. Moreover, an uneven number of gene copies of the *FAD2* gene has also been found in other species such as *G. max*, *G. hirsutum,* and *Z. mays* as compared with Arabidopsis [59,60]. The variation in gene number suggests differential expression of genes showing exclusive expression in one tissue and absent in the other in the same species [61,62]. The phylogenetic clustering of FAD2 proteins based on their genomic origin (A, B, or C) reflects the complex evolutionary history of these genes within the *Brassica* genus (Fig 1).

The gene structure analysis reveals that *FAD2* genes are more prone to structural variations, such as intron retention or loss of UTRs, compared to the more conserved *FAE1* genes (Fig 2a). This variation in gene structure could influence the regulation and functioning of these genes under stressful environments [63]. A comparable pattern of structural features in the *FAE1* and *FAD2* genes has been described for other plant species as well, such as *Phaseolus vulgaris, Glycine max,* and *Sorghum bicolor* [64–67]. The conservation of crucial domains in both FAE1 and FAD2 proteins across species indicates their essential roles in maintaining fatty acid desaturation and elongation processes, despite evolutionary pressures [68,69]. The presence of cis-acting elements linked to signaling agents such as salicylic acid (SA) and jasmonic acid (JA), ABRE and DRE etc suggests that these proteins may direct or indirect role in the stress responsiveness [68,70]. These structural and regulatory predictions suggest that *FAE1* and *FAD2* may contribute to stress-induced lipid remodeling (Fig 3).

Salt stress significantly reduced the growth of the plants in cultivars of both species (*B. napus* and *B. juncea*) as reflected by decreased shoot and root length and fresh and dry biomass in shoots and roots. Although both the species varied in their response towards salinity, the growth in *B. napus* was more depressed by salinity than *B. juncea* (Fig 5). These results were consistent with Kapoor and Pande [71] who reported the reduced shoot length in fenugreek when grown under variable salt stress levels. Another study focusing salt tolerance in *B. napus* reported a profound decrease in shoot and root length when 200 mM salt was applied [72]. Fresh and dry biomass was drastically reduced after the application of salt stress. The shoot fresh weight of both species was more affected at mild salinity as compared to shoot dry weight however at higher salinity levels the shoot fresh weight of *B. juncea* was more affected as compared to shoot dry weight. The opposite trend was observed for *B. napus* at higher salinity level. Root fresh and dry weights were also affected under salt stress however the effect was comparable in both the species. Root fresh weight was more affected at high salt levels as compared to root dry weight under high salt conditions (200 mM). Similar results were found by Jan et al. [73], Ma et al. [74] and [75] where shoot and root biomass production was significantly decreased under high salt concentration in *Brassica*. Contrary to this, a QTL mapping-based study in *B. napus* demonstrated that lines showing slower growth and low biomass production rate were more salt tolerant [76].

The analysis of *FAE1* and *FAD2* gene expression in different tissues of *Brassica* under salt stress is essential to understand how fatty acid metabolism contributes to stress adaptation at a spatial level. Salt stress disrupts cellular homeostasis, causes ionic imbalance, and damages membranes. Since membrane integrity depends largely on fatty acid composition, genes involved in fatty acid elongation and desaturation play a critical role in stress tolerance. *FAE1* is responsible for the elongation of fatty acids to produce very-long-chain fatty acids, which contribute to membrane structure and seed oil composition. *FAD2* catalyzes the conversion of oleic acid to linoleic acid, increasing the degree of unsaturation in membrane lipids. Increased unsaturation enhances membrane fluidity, which is crucial for maintaining membrane stability and function under saline conditions. Salt stress responses are tissue-specific. Roots are the first organs exposed to salinity and are involved in ion uptake and osmotic adjustment, while leaves experience secondary effects such as oxidative stress and altered metabolism. Reproductive tissues directly influence seed development and oil accumulation. Therefore, evaluating the expression of *FAE1* and *FAD2* across different tissues allows the identification of tissue-specific regulatory patterns and reveals whether these genes contribute to adaptive responses in vegetative versus reproductive organs. Furthermore, since *FAE1* is predominantly associated with seed lipid biosynthesis and *FAD2* functions in both vegetative and reproductive tissues, comparing their expression profiles under salt stress can clarify how lipid metabolism is reprogrammed to balance stress tolerance and oil quality. The expression patterns of *FAE1* and *FAD2* genes under salinity stress provided insights into the molecular responses of *B. juncea* and *B. napus* to osmotic challenges. Salt-induced upregulation of *FAE1.1* in specific tissues, particularly in stems and siliques, indicates a targeted response aimed at ensuring the availability of VLCFAs necessary for seed development and energy storage under a stressful environment. The up regulation of *FAE1.1* gene under salt stress depicts its stress-response both in the vegetative and reproductive organs (Fig 6). The differential expression of *FAE1.1* in *Brassica* species under investigation suggests the utilization of distinct regulatory networks to modulate fatty acid elongation in response to salinity [77,78].

The varied expression of *FAD2.1*, especially its upregulation in roots under high salinity, highlights the role of this gene in enhancing unsaturated fatty acid production, which is crucial for maintaining membrane fluidity and function in root cells exposed to osmotic stress (Fig 6). The downregulation of *FAD2.1* in siliques at lower salinity levels, followed by an upregulation at higher salinity (Fig 6b), could indicate a threshold effect where moderate stress does not significantly disrupt fatty acid desaturation, but higher stress levels trigger a compensatory increase in desaturase activity [79–81]. It imlpies that *FAD2.1* gene is responsive to salt stress in the reproductive organs.

Differential tissue-specific expression suggests spatial regulation of fatty acid biosynthesis pathways to balance stress adaptation and developmental processes. The changes in the expression of *FAE1.1* and *FAD2.1* genes under salt stress conditions were also reported earlier [50,64,66,82], implying their significant role in the saline environment. The spatio-temporal expression of *FAE1.1* and *FAD2.1* genes suggests their specific roles in particular organs and tissues [58,69,83,84]. These expression patterns are consistent with the role of *FAE1* and *FAD2* genes in mediating adaptive responses to abiotic factors. The modulation of their expression in different tissues suggests a synchronized response to retain lipid homeostasis across the entire plant body, ensuring that both membrane integrity and energy storage are preserved under challenging environmental conditions. Overall, tissue-specific expression analysis of *FAE1* and *FAD2* under salt stress provides mechanistic insight into membrane adaptation, stress resilience, and oil composition stability in *Brassica*, supporting strategies for developing salt-tolerant and high-quality oilseed varieties.

The yield attributes were also significantly reduced under salt stress in both the Brassicas. However, *B. juncea* showed less reduction in all the observed yield-related parameters except plant height where the percentage reduction was comparable in both the species (Fig 8). It is reported that the accumulation of excessive salt ions in plant cells inhibits the elasticity of the cell wall that causes early cellular rigidity that consequently leads to less cell enlargement followed by reduced plant height [85]. Number of primary branches declined significantly in *B. napus* as compared to *B. juncea* at both NaCl level. A study conducted on various cultivars of *B. juncea* and *B. napus* also reported similar decline in yield attributes like plant height, no of branches per plant, no of siliques, silique length and seed weight in both the studied cultivars under 9 dS m^–1^ NaCl stress [86]. The effect of salinity on silique length and number per plant was also more evident in *B. napus* as compared to *B. juncea*. The number of siliques is considered to be a critical parameter to determine seed yield. Similar trends have been shown where the number of siliques per plant were less affected by salinity in *B. juncea* as compared to *B. napus* [87,88]. Another study in spring canola (*B. napus*) reported a significant reduction in the number of siliques and other attributes under salinity stress [89]. This decrease might be associated with an increase in abscisic acid (ABA) and pollen viability [90]. A decrease in silique length due to salinity is reported in two cultivars of *B. napus* [91].

In this study, a significant reduction in yield under stress was observed in both the Brassicas. However, this reduction was much higher in *B. napus* compared to *B. juncea*. This reduction in yield could be due to the number and length of siliques and the number of primary branches may be the reason behind the poor seed yield per silique and per plant in *B. napus*. These results are in line with the results of other researchers. Salinity stress decreases the number of siliques per stem and the number of seeds per silique leading to a decrease in overall grain yield [92,93]. Overall, the decline in the yield related traits under salinity indicates that molecular alterations such as altered lipid metabolism are reflected at the whole plant level.

Under salt stress, oilseed *Brassica* plants typically exhibit a reduction in both oil content and quality. This decline is often attributed to increased osmotic pressure in the soil solution, imbalances in the nutrients and critical elements, smaller seed size, and diminished cellular metabolic activities, which adversely affect the total oil, lipids, proteins, and fatty acids content [94–96]. Reduction in the oil contents of *Brassica* species has been previously reported [97,98]. To maintain balanced osmotic pressure within plant cells, the presence of soluble sugars and proteins becomes critically important [99]. An upsurge in the total protein content of *B. napus* was reported by [97] while [98] reported a reduction in *B. juncea* under salt stress conditions. The decline in the protein level might be attributed to the disability of the plant to completely utilize nitrogen compounds and the inhibition of nitrogen supply to make amino acids and proteins [100]. In the present study, *B. juncea* displayed a modest decline in oil and a notable upsurge in the protein content compared to the protein content at 100 mM, indicating its superior tolerance and adaptability to saline conditions compared to *B. napus* (Fig 9). This suggests that *B. juncea* is more effective at maintaining oil quality in saline environments.

The findings align with previous studies on *Brassica* and other plant species tolerant to salt stress such as cotton, and safflower [30,101–104], where lipid biosynthesis is often preserved under stress to ensure membrane integrity and energy storage. The lack of significant changes in these parameters could be attributed to the presence of effective osmoregulatory mechanisms in these species, possibly involving the accumulation of compatible solutes like proline, which stabilize proteins and membranes under osmotic stress [105].

In the current study, total SFAs including stearic and palmitic acid exhibited a contrasting trend in *B. juncea* and *B. napus* where an overall increase and decrease were observed for both species, respectively (Fig 10d-e). The increase in saturated fats in *B. juncea* might enhance membrane rigidity, a protective adaptation to counter the fluidizing effect of salt-induced osmotic stress. However, in previous research [97] and [106], a decline in the SFAs content was noticed in favour of increased monounsaturated fatty acids production as a defence strategy against the oxidative damages caused by NaCl stress by restructuring membranes, regulating membrane fluidity and restrict the permeability to Na+ and Cl^−^ ions [107–109]. In some other studies [110] and [111], an increase in the SFAs content was observed under NaCl stress in sunflower and black mustard, respectively. It is speculated that an increased proportion of saturated lipids may result in a gel phase, which may halt the normal functioning of the cell membrane [112]. Erucic acid levels in *B. juncea* remained unchanged while decreased in *B. napus* in the current study (Fig 10a) which is in contrast with the prior research [98]. No substantial changes in the erucic acid level of either salt-tolerant or salt-sensitive cultivars of *B. napus* were observed [101]. Likewise, we did not detect any significant changes in the total unsaturated fatty acid contents (both poly and monounsaturated fatty acids) for both *Brassica* species during this study (Fig 10a-10c, 10f-10g). In contrast, a decrease in the unsaturated fatty acid (UFAs) levels of black mustard has been reported under salinity stress [111]. It may be due to the diminished activities of lipid metabolism enzymes under stressful environments. Reduced amounts of UFAs under salinity stress could be a defense mechanism to counter the oxidative damage caused by Na^+^ and Cl^−^ ions. However, the reduced unsaturated fatty acid index maight also results in reduced membrance fluidity and permeability to salt ions [108,109,113]. The reduction in the amount of UFAs has also been reported in soybean [114]. This resilience to salt stress for different fatty acid contents may be attributed to the strong genetic control due to high heritability and genotypic coefficient of variation of these traits in these *Brassica* species [115,116]. The stability of linoleic acid levels across salinity treatments is noteworthy, as this essential fatty acid plays central role in conserving membrane fluidity and functioning. The tight regulation of linoleic acid biosynthesis, despite environmental stress, suggests that its production is a priority for maintaining cellular homeostasis. Consistent oil, protein and fatty acids composition also points towards the better tolerance mechanism implied in the *Brassica* species helping crop thrive better without compromising on oil contents and quality parameters as reported in our previous studies [55,117], which elaborated enhanced protection of photosystem II in *B. juncea* leading to better photosynthetic performance under 300 mM NaCl levels as well as efficient antioxidant defence system, organic solutes and glucosinolates accumulation. Influence of salt stress was more pronounced in the traits developing at the time of stress and the reduction of those traits significantly contributed to a reduction in the overall wheat yields [118,119]. Therefore the time of salt stress could also be the possible reason for the resilience of oil and protein contents and fatty acid profile observed in the current research study.

Although *FAE1* and *FAD2* exhibited differential transcriptional regulation under salt stress, no significant alteration was detected in erucic acid or overall fatty acid composition. This apparent discrepancy is biologically plausible because fatty acid biosynthesis is regulated at multiple levels beyond transcription. Lipid metabolic pathways are subject to post-transcriptional regulation, enzyme turnover, substrate availability, and feedback control mechanisms that collectively buffer fluctuations in gene expression to maintain membrane and seed oil homeostasis [120–122].

In polyploid species such as *Brassica*, the presence of multiple homologs and functionally redundant desaturase and elongase enzymes may further stabilize metabolic flux, limiting changes in final fatty acid composition despite transcriptional variation in individual genes [123]. Moreover, seed oil composition is a highly canalized trait under strong genetic control, often remaining relatively stable across moderate environmental perturbations [124].

Therefore, the stability of erucic acid and related fatty acids observed in this study likely reflects robust metabolic buffering and compensatory regulation within the lipid biosynthetic network. Rather than indicating a lack of functional relevance, these findings suggest that transcriptional modulation of *FAE1* and *FAD2* under salt stress may contribute to maintaining lipid homeostasis and preserving seed oil quality under saline conditions.

## Conclusion

In summary, this study provides a comprehensive evaluation of the role of *FAE1* and *FAD2* genes in *Brassica* under salt stress through an integrated molecular, physiological, and biochemical approach. Bioinformatics analysis confirmed the conserved structure and functional relevance of these genes in fatty acid metabolism, supporting their potential involvement in stress adaptation. Salt stress imposed at the seedling stage induced transcriptional modulation of *FAE1* and *FAD2*, with tissue-specific expression patterns indicating spatial regulation of lipid metabolic pathways. Although salinity significantly affected growth and yield-related traits, fatty acid composition and erucic acid content remained largely stable, suggesting the presence of robust metabolic buffering mechanisms that preserve oil quality under stress. Collectively, the findings establish a continuum from gene regulation to agronomic performance and seed quality, highlighting the resilience of lipid biosynthetic networks and their importance in maintaining productivity in *Brassica* under saline environments. These insights provide a valuable foundation for future strategies aimed at improving salt tolerance while safeguarding oilseed quality. These findings contribute to our knowledge of stress tolerance mechanisms in *Brassica* species and offer valuable insights for developing more resilient crop varieties with better oil quality through targeted breeding and biotechnological interventions. Furthermore, the study’s findings on gene expression dynamics provide a foundation for exploring gene-editing approaches to augment stress tolerance in *Brassica* crops. Moreover, cultivation of resilient *Brassica* crops on saline lands will also help in the increase of *Brassica* crop production hence lessening the gap between domestic oil production and consumption in Pakistan and saving huge amounts of foreign exchange being spent on the import of edible oil.

## Supporting information

S1 Table Primers used for gene expression studies through qPCR. (PDF)

S2 Table *FAE1* and *FAD2* genes and proteins details from six *Brassica* species as well as Arabidopsis. (PDF)

S3 Table Motif sequences in FAE1 and FAD2 protein sequences. (PDF)
