# Supplementary material for: FAE1 and FAD2 gene expression dynamics and fatty acid modulation in Brassica under salt stress: A molecular insight
Source: PLoS One. 2026 Apr 6;21(4):e0345945. doi: 10.1371/journal.pone.0345945 (PMC13052875; doi:10.1371/journal.pone.0345945)
Supplement: S1 Table — (PDF) [file pone.0345945.s001.pdf]

S1 Table: Primers used for gene expression studies through qPCR

Sr. No.	**Primer Name**	**Sequence (5’-3’)**
**1**	FAE1.1-F	GAAACACTTCATCTAGCTCA
**2**	FAE1.1-R	TTAAAGCCTGACCCTAAAGC
**3**	FAD2.1-F	AGTGGGATTGGTTGAGGG
**4**	FAD2.1-R	ATCGTGGAGAACAGATGATG
**5**	ACT-F	TGTGACAATGGAACTGGAAT
**6**	ACT-R	GACCCATCCCAACCATGA
