# Supplementary material for: FAE1 and FAD2 gene expression dynamics and fatty acid modulation in Brassica under salt stress: A molecular insight
Source: PLoS One. 2026 Apr 6;21(4):e0345945. doi: 10.1371/journal.pone.0345945 (PMC13052875; doi:10.1371/journal.pone.0345945)
Supplement: S2 Table — (PDF) [file pone.0345945.s002.pdf]

S2 Table: *FAE1* and *FAD2* genes and proteins details from six *Brassica* species as well as Arabidopsis

Gene ID	Gene Name	Chr. No.	Strand	Location	Transcript length (bp)	Protein length (aa)	Mol Wt (Da)	pI	GRAVY	Subcellular localization
AT3G12120.1	AtFAD2	3	-	3860445- 3862880	1152	383	44047.75	8.39	-0.094	Endoplasmic reticulum
BolC5t34237H	BolFAD2.1	C05	+	49254545-49257041	1374	457	52726.58	9.21	-0.243	Endoplasmic reticulum, Plasma membrane
BolC1t05264H	BolFAD2.2	C01	+	47179210-47180364	1155	384	43902.54	8.55	-0.128	Endoplasmic reticulum
BniB01g052830.2N	BniFAD2	B01	+	53287821-53288975	1155	384	44216.84	8.25	-0.136	Endoplasmic reticulum
BraA05g035880.3.5C	BraFAD2	A05	+	24683213-24686066	1374	457	52733.53	9.2	-0.263	Endoplasmic reticulum, Plasma membrane
C05p55690.1_BnaDAR	BnaFAD2.1	C05	+	51452170-51454696	1374	457	52724.61	9.21	-0.234	Endoplasmic reticulum, Plasma membrane
A05p37230.1_BnaDAR	BnaFAD2.2	A05	+	36891425-36894202	1410	469	54151.19	9.2	-0.309	Endoplasmic reticulum
BjuVB01G42140	BjuFAD2.1	B01	+	50639650-50642532	1155	384	44232	8.53	-0.128	Endoplasmic reticulum
BjuVA05G37350	BjuFAD2.2	A05	+	33916717-33919510	1374	457	52720.53	9.2	-0.256	Endoplasmic reticulum, Plasma membrane
BcaB06g25543	BcaFAD2.1	B06	+	1656514-1657668	1155	384	44231.96	8.53	-0.128	Endoplasmic reticulum
BcaC05g29288	BcaFAD2.2	C05	+	56181536-56182690	1155	384	44172.85	8.53	-0.121	Endoplasmic reticulum
BcaC09g48213	BcaFAD2.3	C09	-	4449770-4450924	1155	384	43989.66	8.66	-0.143	Endoplasmic reticulum
AT4G34520	AtFAE1	4	+	16494205-16495842	1521	506	56264.03	9.4	-0.052	Plasma membrane
BolC3t20858H	BolFAE1	C03	-	67251496-67253016	1521	506	56455.03	9.3	-0.128	Plasma membrane
BniB03g020700.2N	BniFAE1	B03	+	9135884-9140082	2907	968	108781.93	9.35	-0.065	Plasma membrane
BraA08g016330.3.5C	BraFAE1	A08	-	13006381-13007901	1521	506	56369.88	9.26	-0.117	Plasma membrane
A08p16760.1_BnaDAR	BnaFAE1.1	A08	-	16402673-16404193	1521	506	56456.06	9.26	-0.1	Plasma membrane
C03p85720.1_BnaDAR	BnaFAE1.2	C03	-	67615312-67616827	1428	475	52938.26	9.39	-0.052	Plasma membrane
BjuVB03G19720	BjuFAE1.1	B03	+	9061749-9063269	1521	506	56404.02	9.34	-0.103	Plasma membrane
BjuVA08G16890	BjuFAE1.2	A08	-	13383605-13385125	1521	506	56383.9	9.26	-0.117	Plasma membrane
BcaB03g11828	BcaFAE1.1	B03	-	1783278-1784798	1521	506	56438.04	9.34	-0.105	Plasma membrane
BcaC01g00899	BcaFAE1.2	C01	-	10237290-10238810	1521	506	56367.86	9.26	-0.129	Plasma membrane
