# Supplementary material for: FAE1 and FAD2 gene expression dynamics and fatty acid modulation in Brassica under salt stress: A molecular insight
Source: PLoS One. 2026 Apr 6;21(4):e0345945. doi: 10.1371/journal.pone.0345945 (PMC13052875; doi:10.1371/journal.pone.0345945)
Supplement: S3 Table — (PDF) [file pone.0345945.s003.pdf]

S3 Table: Motif sequences in FAE1 and FAD2 protein sequences

Motif No	Sequence in FAE1	Sequence in FAD2
1	RSTLHRFGNTSSSSIWYELAYIEAKGRMKKGNKVWQIALGSGFKCNSAVW	MPHYHAMEATKAIKPILGEYYQFDGTPVVKAMWREAKECIYVEPDRQGEK
2	CDDSSWLDFLRKIQERSGLGDETHGPEGLLQVPPRKTFAAAREETEQVII	WKYSHRRHHSNTGSLERDEVFVPKKKSDIKWYGKYLNNPLGRTVMLTVQF
3	MVSNCLFRVGGAAILLSNKPRDRRRSKYELVHTVRTHTGADDKSFRCVQQ	TYLQHTHPSLPHYDSSEWDWLRGALATVDRDYGILNKVFHNITDTHVAHH
4	FTVFGSVLYIATRPKPVYLVEYSCYLPPTHCRSSISKVMDIFYQVRKADP	WPLYWACQGCVLTGVWVIAHECGHHAFSDYQWLDDTVGLIFHSFLLVPYF
5	FNLGGMGCSAGVIAIDLAKDLLHVHKNTYALVVSTENITYNIYAGDNRSM	GGFACHFHPNAPIYNDRERLQIYISDAGILAVCYGLYRYAAAQGVASMVC
6	MGKKLFKDKIKHYYVPDFKLAIDHFCIHAGGRAVIDVLEKNLGLAPIDVE	MGAGGRMQVSPPSKKSETDTJKRVPCETPPFTVGELKKAIPPHCFKRSIP
7	FKNTNVNPKDIGILVVNSSMFNPTPSLSAMVVNTFK	RSFSYLIWDIIIASCFYYVATTYFPL
8	YHYVITNLFNLCFFPLTAIVAGKAYRLTIDDLHHLY	MSLNRLTRAPVNLNGLPSAQSHLTRTREIHYQRDREREKER
9	DDENGKTGVSLSKDITDVAGRTVKKNIATLGPLILP	GWPLYLAFNVSGRP
10	NVKASTNSPWEHCIDRYPVKIDSDSGKSETRVQNGR	GVPLLIVNGFLVLI
11	YLQHNLITIAPLFA	KGVFWYNNKL
12	SEKLLFFVTF	RQRESLRRSFFVGFIVINVKSSSPPT
13	RAFNCVKQEK	MEGRPCKEER
14	MDDNNPIRKN	
15	WEHCIDRLPV	
